# Antimicrobial Properties of Chitosan-Modified Cotton Fabric Treated with Aldehydes and Zinc Oxide Particles

**DOI:** 10.3390/ma16145090

**Published:** 2023-07-19

**Authors:** Desislava Staneva, Daniela Atanasova, Daniela Angelova, Petar Grozdanov, Ivanka Nikolova, Ivo Grabchev

**Affiliations:** 1 Department of Textile, Leather and Fuels, University of Chemical Technology and Metallurgy, 1756 Sofia, Bulgaria; d.atanasova1@abv.bg (D.A.);; 2 The Stephan Angeloff Institute of Microbiology, Bulgarian Academy of Sciences, 1756 Sofia, Bulgaria; grozdanov_bg@yahoo.com (P.G.); vanianik@mail.bg (I.N.); 3 Faculty of Medicine, Sofia University “St. Kliment Ohridski”, 1407 Sofia, Bulgaria

**Keywords:** composite, chitosan, aldehydes, zinc oxide, cotton fabric, antimicrobial

## Abstract

Chitosan is a natural biopolymer with a proven ability to impart textile materials with antimicrobial properties when loaded onto them. The mechanism of its bacteriological activity depends on the contact between the positive and negative charges of the amino groups located on the surface of the microbes. Unfortunately, the type of microorganisms and pH influence this action–shortcomings that can be avoided by chitosan modification and by loading its film with substances possessing antimicrobial properties. In this study, chitosan was modified with benzaldehyde and crosslinked with glutaraldehyde to form a film on the surface of cotton fabric (CB). Also, another material was obtained by including zinc oxide particles (CBZ) synthesized in situ into the chitosan coating. The performed analyses (contact angle measurement, optical and scanning electron microscopy, FTIR, XRD, and thermal analysis) evidenced the modification of the cotton fabric and the alteration of the film properties after zinc oxide inclusion. A comparison of the antimicrobial properties of the new CB with materials prepared with chitosan without benzaldehyde from our previous study verified the influence of the hydrophobicity and surface roughness of the fabric surface on the enhancement of antimicrobial activity. The microbial growth inhibition increased in the following order: fungal strain Candida lipolytica >Gram-positive bacteria Bacillus cereus >Gram-negative bacteria Pseudomonas aeruginosa. The samples containing zinc oxide particles completely inhibited the growth of all three model strains. The virucidal activity of the CB was higher against human adenovirus serotype 5 (HAdV-5) than against human respiratory syncytial virus (HRSV-S2) after 60 min of exposure. The CBZ displayed higher virucidal activity with a Δlog of 0.9 against both viruses.

## 1. Introduction

The demands for textiles with properties meeting the requirements for utilization in healthcare and medical practice are related to the changing attitudes of modern humans towards wellbeing, comfortable living, and the awareness of the vital balance between the human microbiome and the environment [1]. Various antimicrobial coatings have been applied to protect textiles from the attack of microorganisms. The obtained materials reduce the dangerous possibility of further post-trauma infections and the spread of diseases. It also eliminates the appearance of unpleasant odours and stains, to which fabrics of natural fibres are especially susceptible [2]. Although different chemicals imparting antimicrobial activity to textiles are used in industrial practice, many of them are toxic to humans and do not degrade easily in nature [3]. On the other hand, the microorganisms themselves develop resistance to widely exploited antimicrobial substances [4].

Therefore, the constant demand for textile industry specialists to develop ecological technologies for an effective antimicrobial defence without harmful chemicals continues [5]. To prevent the rapid spread of microorganisms, it is crucial to understand their interaction with the surface of textile materials and their passing through the stages of attachment, growth, proliferation, biofilm formation, and the ability to spread infections. All microbes (bacteria, fungi, and viruses) have diverse specific structures and pathological behaviour. They possess different mechanisms for attaching to the surface and subsequently causing infections. From this comes the difficulty in obtaining a universal antimicrobial surface. The surface of a textile material can be modified by various physical and chemical methods. Thus, many properties, such as wettability, surface topology, and surface charge, can be altered. Since the mechanism of antimicrobial action is not yet fully understood, it is worth investigating how the change in different surface characteristics affects the antimicrobial properties of the particular textile.

Recently, antimicrobial polymers have been investigated intensively as potential compounds to combat multidrug-resistant pathogens [6]. A typical antimicrobial polymer consists of cationic groups that enable it to adsorb onto negatively charged bacterial membranes. The presence of hydrophilic groups affects the interaction of these polymers with the environment in which they are found, and their hydrophobic substituents penetrate the bilayer lipid membrane of bacteria and disrupt it. The addition of antimicrobial agents via physical or chemical bonds into the polymeric structure may modulate its activity [7]. Polymers’ application for textile modification aims at forming an anti-biofouling and microbicide protection covering [8]. After deposition on the fabric, the macromolecules can be organized in different structures either as a smooth/rough film or polymer brushes [9]. The obtained surfaces can have a variety of organizations: patterned, functionalized, superhydrophobic/superhydrophilic, and smart repulse abilities [10]. Therefore, the type of modification is responsible for the observed antimicrobial mechanism. The limited contact area, the possible penetration and rupture of microbial cells, and its repulsion or slippery capabilities are some factors that determine the anti-biofouling and microbicidal activity of the obtained materials.

Being nontoxic, chitosan is a highly applied biopolymer for textile modification in the form of an antimicrobial coating [11,12]. As a cationic polymer in lower pH media and due to its electrostatic interaction with bacterial cell walls, it possesses good antimicrobial activity resulting in the growth inhibition and death of fungi, bacteria, and yeasts. Hydrophobic chitosan derivatives have also attracted interest in recent years because of their altered interaction with water and their self-organization [13]. Compared with natural chitosan, the chitosan substituted with hydrophobic structures has been found to exhibit greater antimicrobial activity against Gram-positive bacteria, while no significant change has been observed against Gram-negative bacteria [14]. The reason might be that in a neutral environment, chitosan is deprotonated and thus interferes with its interaction with the bacterial cell wall. The presence of hydrophobic substituents in its backbone facilitates binding to the bacterial cell wall. Although chitosan has good film-forming properties, it is necessary to modulate the resulting coating, so that the desired bacteriological efficiency with consumer and environmental satisfaction can be achieved. Through chemical crosslinking, it can be fixed onto the surface of the textile fabric [15,16]. The presence of many amino and hydroxyl groups in the backbone of chitosan facilitates its excellent chelating properties and different antimicrobial action modes at a higher pH. The ability of chitosan to form complexes with zinc ions was used in our previous study to control the size, structure, and distribution of zinc oxide particles over the fibres’ surface [17]. The organic–inorganic nanocomposite thus obtained, combining natural polymers with metals or metal oxides, had an amended structure and responded to external stimuli. The interaction of ZnO particles with the functional groups of chitosan affects the film surface and its ability to swell in water and thus affects its sorption properties. The extremely good antibacterial action against a wide range of microorganisms of ZnO nanoparticles is well known, and they find various applications in everyday life, such as in cosmetics, medical devices, etc. [18,19]. Their advantage is the selective toxicity to bacteria and minimal effect on human cells [20,21]. Recently, the investigation has been focused on the antiviral efficiency of ZnO nanoparticles, and it has been established that their presence is responsible for viral load decreases [22,23]. Jana et al. found that the incorporation of the in situ synthesized ZnO particles into a polymer structure of chitosan or into phenyloxy-functionalized chitosan improved their stability and effective antiviral activity toward human cytomegalovirus [24].

This work aims to obtain a cotton fabric loaded with a polymer film possessing suitable characteristics to provide good antimicrobial properties. The modification of chitosan with benzaldehyde and its crosslinking with glutaraldehyde intend to incorporate new functional groups on the fabric surface to influence its contact with the microorganisms. Including zinc oxide particles in the chitosan layer is expected to change the structure of the resulting coating and achieve a synergistic effect increasing the antimicrobial properties against the various tested types of microorganisms (Gram-positive and Gram-negative bacteria, fungi, and viruses).

## 2. Materials and Methods

### 2.1. Materials

A bleached plain-woven 100% cotton fabric with a surface weight of 135 ± 5 g/m^2^ was used throughout the work. Chitosan with a molecular weight ranging from 600,000 to 800,000 g mol^−^^1^ was purchased from Acros Organics (Geel, Belgium). Zn(NO_3_)_2_ × 6H_2_O and NaOH were purchased from Valerus Ltd. (Sofia, Bulgaria). Glacial acetic acid, benzaldehyde, and glutaraldehyde (25% aqueous solution) were used without further purification as obtained from Merck (Darmstadt, Germany). Distilled water and ethanol were used as solvents.

### 2.2. Preparation of Composite Materials

#### 2.2.1. Preparation of the Solution for Fabric Treatment

Solutions 1 and 2 were prepared according to a procedure described previously [17].

1. The chitosan (2.7% *w*/*v*) was dissolved in water under stirring, with the gradual addition of glacial acetic acid (1% *v*/*v*) to obtain a clear viscous solution.
2. The chitosan (2.7% *w*/*v*) and Zn(NO_3_)_2_ × 6H_2_O (2.7% *w*/*v*) were dissolved in water with the gradual addition of glacial acetic acid (1% *v*/*v*) to obtain a clear viscous solution.
3. A solution of benzaldehyde in ethanol at a concentration of 90μL/mL was prepared.
4. Solution (3) was added to solution (1) at 60 °C, and stirring continued for 2 h.
5. Solution (3) was added to solution (2) at 60 °C, and stirring continued for 2 h.

#### 2.2.2. Cotton Fabric Treatment

The two composite textile materials obtained were named CB and CBZ.

6. Sample CB

The used method was pad–dry–pad–dry–wash.

The cotton fabric was saturated with solution (4), using a liquor to goods ratio of 5:1. After drying, the fabric was infused with a water solution of glutaraldehyde (10% *w*/*w* to chitosan) and dried again at room temperature for 24 h. Subsequently, the sample was rinsed with distilled water and left to dry at room temperature.

7. Sample CBZ

The used method was pad–dry–pad–dry–pad (alkali)–cure–wash.

The cotton fabric was impregnated with solution (5) at a liquor to goods ratio of 5:1, following the same procedure as the CB sample. Afterward, the CBZ sample was immersed in a sodium hydroxide solution with an excess of ten times the stoichiometric amount of zinc ions and thermally treated at 80 °C for 30 min. The resulting composite materials were washed with distilled water and left to dry at room temperature.

In order to remove the unreacted amount of benzaldehyde, the composite materials were washed three times with ethanol.

### 2.3. Characterization

The weight change of the fabric following treatment was determined using the Formula (1):

Percentage change in weight = [(W − Wo)/Wo] × 100,(1)

where Wo and W represent the weights of the fabric before treatment and after immersion in distilled water and subsequent drying, respectively.

Infrared (IR) analysis was conducted using an infrared Fourier transform spectrometer (IRAffinity-1, Shimadzu, Kyoto, Japan) equipped with a diffuse-reflectance attachment (MIRacle Attenuated Total Reflectance Attachment). Measurements were performed within the spectral range of 600–4000 cm^−^^1^. Simultaneous thermogravimetric analysis (TGA) and differential thermal analysis (DTA) were carried out on an STA PT1600 TG-DTA/DSC analyser (LINSEIS Messgeräte GmbH, Selb, Germany) with a heating rate of 10 °C/min, covering the temperature range from room temperature to 600 °C in an air atmosphere.

The pristine cotton fabric, as well as the CB and CBZ, were examined using an optical microscope B-290TB, (Optika ^®^, Ponteranica, Italy). The magnification of the images was set at 40× and 100×. The surface morphology of the composite materials and the formation of ZnO particles were analysed using SEM-EDX equipment (SEM/FIB LYRA I XMU SEM (TESCAN)), operated with a tungsten heating filament. The instrument provided a resolution of 3.5 nm at 30 kV, with an accelerating voltage range of 200 V to 30 kV. Prior to imaging, the investigated samples were coated with gold using a DC magnetron sputtering Au K500X (Quorum Technologies, Lewes, UK). Contact angle measurements of the fabric surface were conducted using a Mobile Surface Analyser–MSA Flexible Liquid (KRÜSS GmbH, Hamburg, Germany). The surface free energy was determined using test liquids: deionized water as a polar liquid and diiodomethane as a nonpolar liquid. The polar and dispersive components of the surface free energy, along with their sum, are presented in Table 1. The reported results for both newly prepared materials, CB and CBZ, represent the average value of five measurements. The composite materials, CB and CBZ, were analysed with an Empyrean Powder X-ray diffractometer (Malvern Panalytical, Almelo, The Netherlands) at 25 °C with an operating voltage and current of 40 kV and 40 mA, respectively. The X-ray diffraction data were recorded by using Cu Kα radiation (λ = 1.5406 Å) in the 2–80° 2θ range, step 0.013°, and a PIXcel3D detector.

### 2.4. Antimicrobial Assay

#### 2.4.1. Antibacterial and Antifungal Assay

The antimicrobial activity of the CB and CBZ was tested by inhibition of the model microbial strains’ growth: Gram-positive *Bacillus cereus*, ATCC 11778, Gram-negative *Pseudomonas aeruginosa*, 1390, and the yeasts *Candida lipolytica* 7618. Microorganisms were obtained from collection of the Institute of Microbiology, Bulgarian Academy of Sciences, Sofia, Bulgaria. The fabric samples with a square shape (10 mm × 10 mm) were placed in test tubes with a sterile meat–peptone broth (MPB) medium (Sigma-Aldrich, Darmstadt, Germany). As controls, test tubes without fabric and those with the untreated starting cotton fabric were prepared. The tubes were inoculated with an equal amount of suspension of each microbial culture and incubated for 24 h at 28–30 °C. The microbial growth was determined by measuring the optical density of the medium at 600 nm (OD600). The antimicrobial activity of the samples was calculated by the reduction in the microbial growth in the presence of the treated fabric samples compared to the control samples.

#### 2.4.2. Virucidal Assay

We conducted the virocidal effect test on the fabrics dyed with the newly synthesized compounds, namely CB and CBZ, using the following approach: Identical pieces of textiles measuring 1 cm^2^ were cut and immersed into a viral suspension (200 µL) containing HAdV5 (106.2 CCID_50_, VR-1516™ ATCC, Manassas, VA, USA) and HRSV-S2 (105.5 CCID_50_, A2 (105.5 CCID50, VR-1540™ ATCC, Manassas, VA, USA) for 30 and 60 min, respectively. As a control, we used nonmodified textiles. The virus suspension was then recovered through exhaustion after the specified time had elapsed. Next, human epithelial type2 (HEp-2, ATCC CCL-23™, Manassas, VA, USA) monolayer cells were inoculated in 96-well plates (Costar^®^, Corning Inc., Kennebunk, ME, USA) and incubated in a humidified atmosphere at 37 °C and 5% CO_2_ for 48 h for HAdV-5 and 72 h for HRSV-2. Following microscopic evaluation, the maintenance medium was removed, and the cells were washed. Subsequently, 0.1 mL of maintenance medium supplemented with 0.005% neutral red dye (N-3246, Invitrogen™, Thermo Fisher Scientific Corporation, St. Bend, OR, USA) was added to each well, and the cells were incubated at 37 °C for 3 h.

After the incubation, the neutral red dye was removed, and the cells were washed once with PBS (20012027, Thermo Fisher Scientific Corporation, St. Bend, OR, USA) Then, 0.15 mL of desorb solution (1% glacial acetic acid and 49% ethanol in distilled water) was added to each well. The optical density (OD) of each well was measured at 540 nm using a microplate reader (Biotek Organon, West Chester, West Chester, PA, USA). The residual infectious virus content was determined using the end-point dilution method, followed by evaluating the Δlogs by comparing each sample with the control.

## 3. Results and Discussion

### 3.1. FTIR Characterization of the Composite Materials

Infrared spectroscopy analysis demonstrated the chemical modification of the chitosan with the aldehydes and its interaction with the zinc oxide particles.

**Figure 1:**
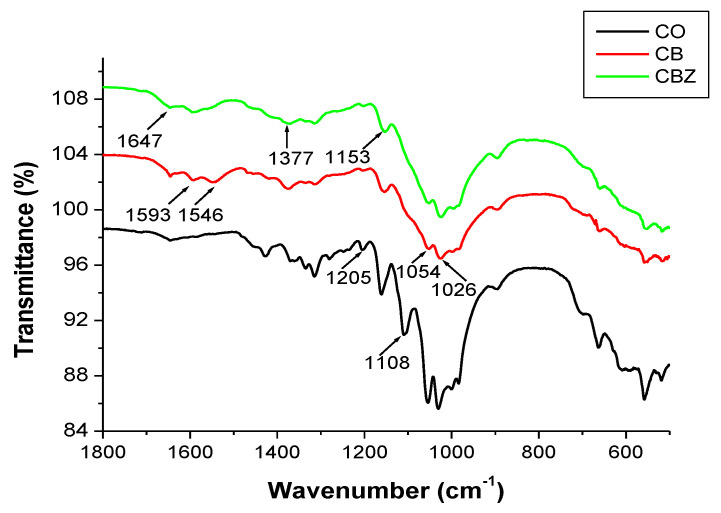
FTIR spectra of the initial cotton fabric (CO) and the CB and CBZ.

Figure 1 presents the IR spectra of the initial cotton fabric (CO) and composite materials CB and CBZ in the 1800 cm^−1^ to 500 cm^−1^ region, which contains peaks characterizing the modification of chitosan with benzaldehyde and with glutaraldehyde and interaction with the ZnO particles. Cellulose and chitosan have similar chemical structures, and their signals in the IR spectrum appear in the same region. Table 2 and Figure 1 show the values of the main bands assigned to cellulose and chitosan, and their corresponding functional groups [25,26].

The comparison of the spectrum of the cotton fabric and those of the CB and CBZ (Figure 1) showed the appearance of new bands at 1647 cm^−^^1^ and 1593 cm^−^^1^. These bands can be assigned to the vibrational oscillation of C=O in the amide groups and of C=N in the Schiff bases, obtained as a result of modifying the chitosan with benzaldehyde and its crosslinking with glutaraldehyde, respectively. The band at 1546 cm^−^^1^ due to deformation of the N-H bond and the band at 1377 cm^−^^1^ due to the C-N of aryl azomethine were observed in the spectrum of the CB. Compared with the spectrum of the CBZ, the first band at 1546 cm^−^^1^ was absent, and the intensity of the second band at 1377 cm^−^^1^ decreased, indicating that chitosan interacted with the ZnO particles.

### 3.2. Thermogravimetric Analysis of the Cotton Fabric and the CB and CBZ Composite Materials 

**Figure 2:**
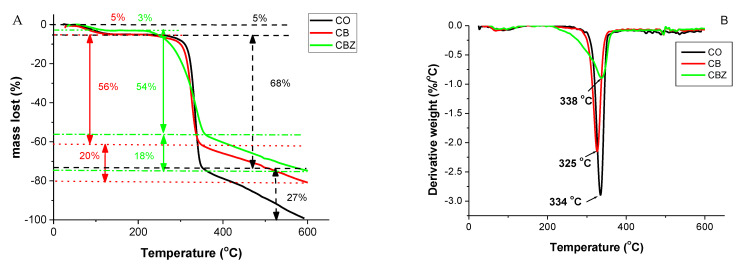
Untreated cotton fabric (CO) and the CB and CBZ samples: (**A**) TGA and (**B**) DTG.

The thermogravimetric analysis (TGA) and its first derivative–the differential thermogravimetric (DTG) curve of the cotton fabric and the CB and CBZ are presented in Figure 2A,B, respectively. A three-stage weight loss was observed for all samples, but the main thermal degradation occurred most significantly at the second stage. The first weight loss started at around 120 °C and was due to moisture loss (3% for the CBZ sample and 5% for the CO and CB samples). During the following temperature range, no change in the samples’ weight was observed until the beginning of their most significant thermal degradation. This stage started at a lower temperature for the CBZ sample, approximately at 210 °C, while for the CB and CO materials, the degradation onset was at about 260 °C (Figure 2A,B). At this stage, the weight loss was due to processes such as the dehydration of the glucosidic units, depolymerization, and degradation [27]. Figure 2B shows that the significant thermal degradation process was at 325 °C for the CB, at 334 °C for the CO, and at 338 °C for the CBZ. For the untreated cotton, the change in weight during this stage was 68%, while for the CB, it was 56%, and for the CBZ, it was 54%. The CBZ lost weight more smoothly than the other materials. Therefore, covering the cotton fabric with a layer of chitosan modified with benzaldehyde and crosslinked with glutaraldehyde, along with the addition of zinc oxide particles, changed the fibres’ degradation processes. The third stage started at about 350 °C, and the weight change in all three samples proceeded uniformly, with no inflection point observed in the graphs, presented in Figure 2B. At this stage, thermo-oxidative processes took place, leading to the formation and release of volatile products. At 600 °C, the cotton fabric burned completely without a dry residue. The largest dry residue of 25% remained from the CBZ and 19% from the CB. Therefore, coating the cotton fabric with aldehydes-modified chitosan and incorporating ZnO particles shifted the thermal degradation of the composite material to a lower temperature, but melting the coating on the fibres’ surface slowed down the degradation process and protected them from further degradation.

**Figure 3:**
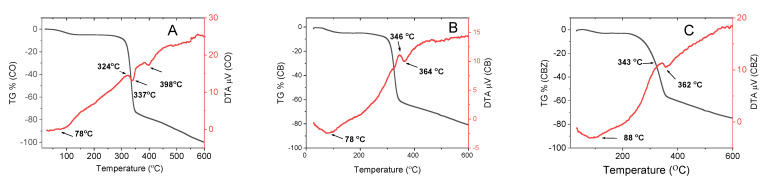
Comparison between the TG and DTA curves: (**A**) cotton fabric; (**B**) CB; (**C**) CBZ.

Figure 3 presents the comparison of the differential thermal analysis (DTA) with the thermogravimetric curves of the pristine cotton fabric and the composite materials. With the increase in the temperature, an endothermic process was observed in all three samples because of the moisture release, which had a more pronounced minimum for the CB at (78 °C) and for the CBZ (at 88 °C). Next, the heating continued with the exothermic process. For the cotton fabric, this process lasted up to a temperature of 324 °C; after that, an endothermic process started, corresponding to the significant change in the weight of the samples determined by thermogravimetric analysis. The destruction continued with an exothermic process followed by a second endothermic process, not observed in the case of the CB and CBZ. Their first endothermic minimums were followed by exothermic processes, which continued to a higher temperature than the cotton–to 346 °C and 343 °C, respectively. Then an endothermic process began, with minimums at 364 °C and at 362 °C, respectively, and the destruction continued with exothermic processes.

### 3.3. Morphological Properties of the Composites

#### 3.3.1. Optical Microscope Observation

**Figure 4:**
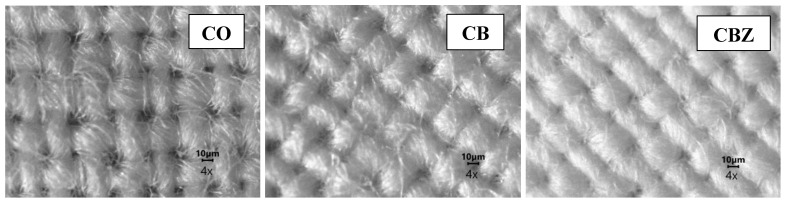
Optical microscope photographs of the cotton fabric (CO) and the (CB) and (CBZ) at a 40× magnification.

Figure 4 shows the optical microscope images of the cotton fabric and CB and CBZ, observed with objective 4×. In the untreated cotton fabric, the individual fibres in the yarn, the weave of the fabric, and the gaps formed during weaving were visible. The CB obtained by applying benzaldehyde and glutaraldehyde-modified chitosan had the structure and the voids produced during weaving still visible, but the resulting layer bonded the individual fibres and filled some of the gaps. The insertion of zinc oxide particles into the chitosan film covering the cloth resulted in a denser layer over the CBZ sample. The effect of the coating was the filling of the micro and macro gaps in the tissue structure.

**Figure 5:**
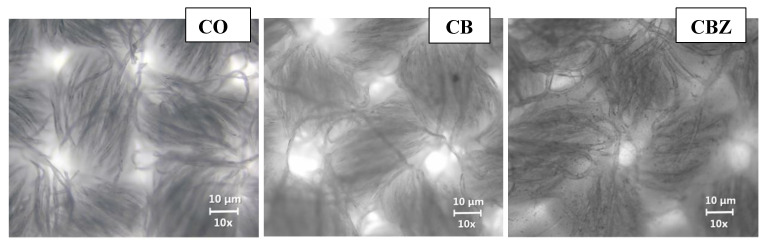
Optical microscope photographs of the cotton fabric (CO) and the (CB) and (CBZ). (The pictures were taken at 100× magnification using lighting from below).

Figure 5 shows photographs of the materials at a higher magnification 100×, observed with objective 10× and illuminated from below. Under these conditions, no pronounced difference between the structure of the original cotton fabric and that of the CB was observed. The resulting chitosan layer was transparent and formed mainly on the fibres. In the CBZ, the film was more pronounced as the small-scale black dots of the zinc oxide particles were scattered throughout it. The optical microscope photographs of both the CB and CBZ showed that the obtained coatings on the cotton fabric surface were uniform. Also, zinc oxide particles were distributed evenly in the structure of the chitosan layer, visible from the optical microscope photographs at a higher magnification.

#### 3.3.2. SEM Analysis

**Figure 6:**
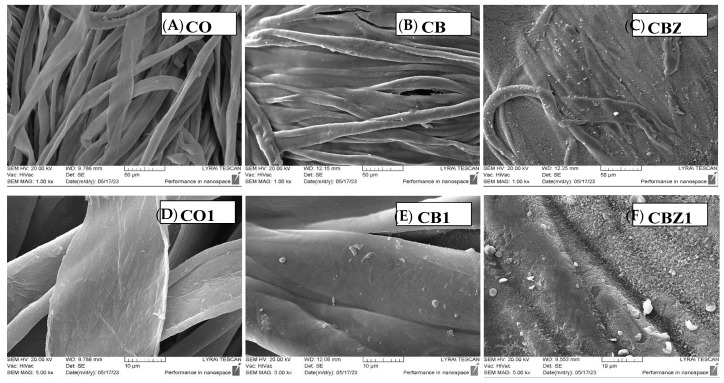
SEM images of the samples: at 1000× magnification: (**A**) CO; (**B**) CB; (**C**) CBZ; at 5000× magnification: (**D**) CO1; (**E**) CB1; (**F**) CBZ1.

SEM analysis was used to establish more precisely the structure of the layers formed on the fabric surface. Figure 6A,D show the typical form and surface of cotton fibres (CO), which looked like twisted bands and had an uneven surface, formed by the relatively well-aligned fibrils to the fibber axis. The modified chitosan with benzaldehyde tended to form a layer on the fibres’ surface with a bumpy structure and with aggregates of different sizes that occurred during the drying process due to the interaction of benzene rings, as seen in Figure 6B,E. The inclusion of ZnO particles densified the formed layer and tightly covered the fibres (Figure 6C). Numerous relatively well-distributed particles were observed on its surface. As seen at a higher magnification, the layer surface texture had high porosity (Figure 6F).

**Figure 7:**
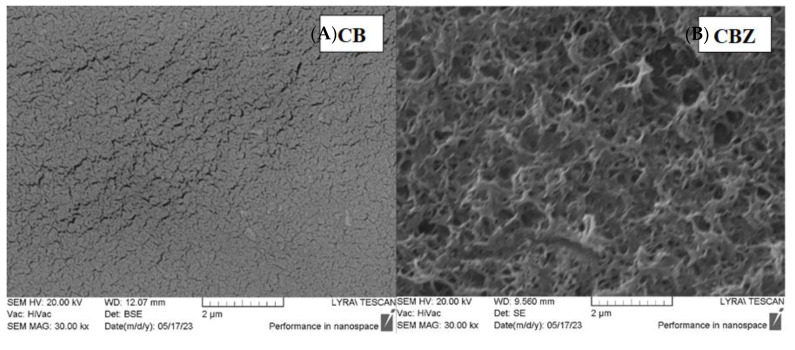
SEM images at 30,000× magnification of (**A**) CB and (**B**) CBZ.

At a higher magnification of 30,000×, shown in Figure 7A, the surface morphology of the modified samples was more clearly visible. Sample CB had a rough structure composed of interconnected capillaries, formed as a result of the modification with aldehydes and the presence of benzene nuclei as substituents in the main chain of chitosan. This typical surface, also observed in another study, has been explained by the interruption of the polymeric chains arrangement that results from the coupling of the chitosan amine groups with the aldehydes [28]. The incorporation of ZnO into chitosan film changes its surface into a highly developed foam-like porous structure, as seen in Figure 7B. A large amount of zinc ions used in the CBZ preparation, affected the shape and distribution of zinc oxide on the surface of CBZ. As a result, star-shaped nanoparticles were observed, which were interconnected and formed numerous well-distributed pores inside the chitosan film.

**Figure 8:**
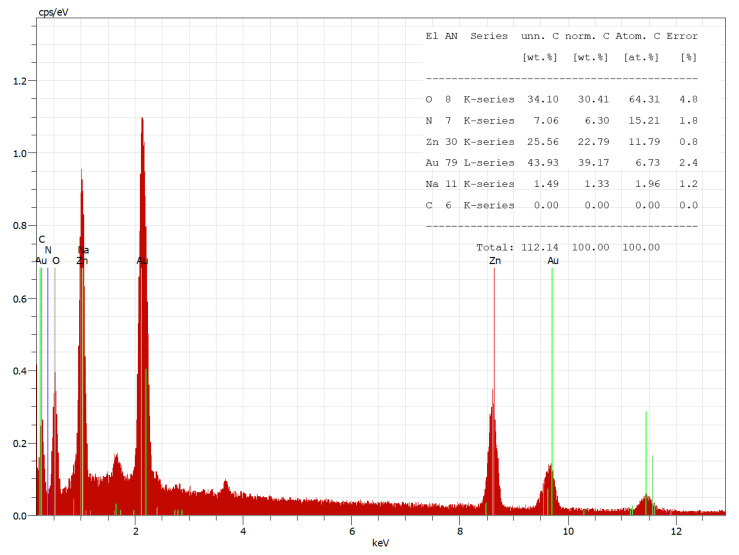
Energy-dispersive X-ray (EDX) spectrum of the CBZ.

In Figure 8, the EDX spectrum of the CBZ shows the presence of zinc peaks, and the calculated atomic percent for Zn was 11.79%.

#### 3.3.3. Contact Angle Measurement

**Figure 9:**
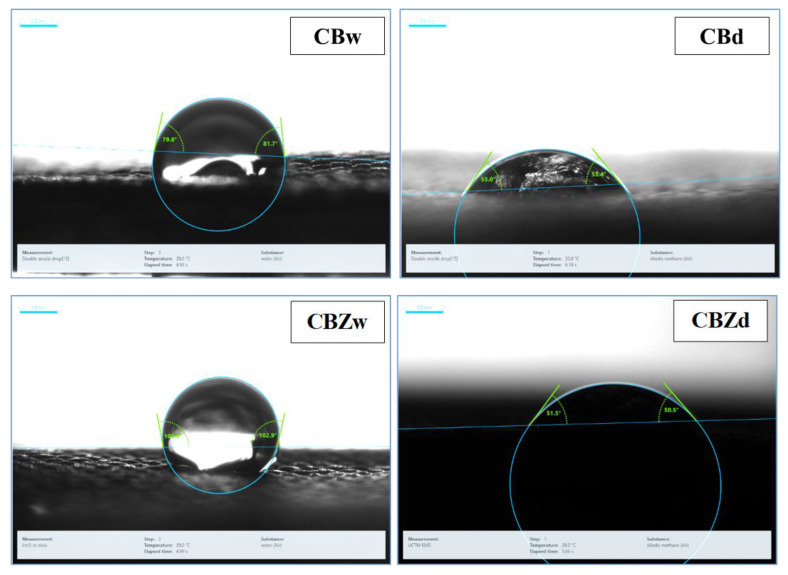
Contact angle measurement: (CBw) water on CB; (CBd) diiodomethane on CB; (CBZw) water on CBZ; (CBZd) diiodomethane on CBZd.

Figure 9 shows pictures of a drop of water and a drop of diiodomethane on the CB and CBZ. The average values obtained from five measurements of the contact angle and free surface energy are presented in Table 3. Modification with benzaldehyde and glutaraldehyde engaged the polar amino groups of chitosan in the Schiff base formation and introduced new nonpolar benzene groups on the material surface. The measured contact angle with a drop of water was 77.13°; while in diiodomethane, it was 53.30°. For the CBZ, the contact angle of water (101.76°) was significantly larger than on the CB; correspondingly, the surface energy was smaller. That means the surface of the CBZ material, in addition to containing nonpolar groups, was also rougher, which affected the contact of the droplet with the surface of the material. Thus, the contact angle increased and the total surface free energy decreased.

The lower surface energy was mainly due to the lower polar component and the strong polarized interaction of the hydrogen bonds, indicating the presence of fewer polar groups over the material surface owing to their interaction with zinc oxide particles. The dispersed component in the CBZ was slightly larger than that in the CB. That corresponds to the van der Waals forces, which increased due to a change in the interaction of nonpolar groups in the structure of the surface layer.

#### 3.3.4. X-ray Diffraction (XRD)

**Figure 10:**
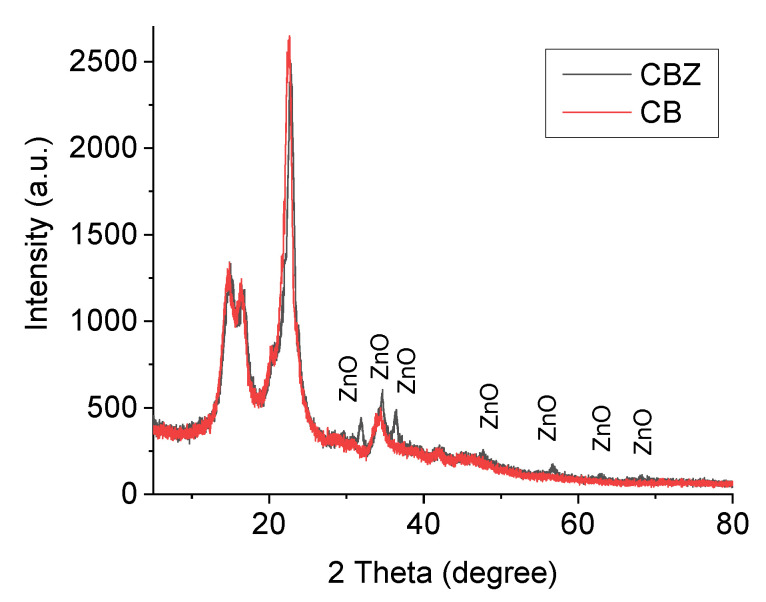
X-ray diffraction spectra of the CB and CBZ.

The X-ray patterns of the CB and CBZ are compared and shown in Figure 10. Diffraction peaks at values of 2θ: 31.94, 34.69, 36.67, and 56.70 degrees were well visible in the spectrum of CBZ, and with less intensity, there were peaks at: 47.70, 63.07, and 68.09 degrees. According to the literature data, these peaks corresponded to a hexagonal crystal structure of the synthesized ZnO particles [29].

### 3.4. Antimicrobial Activity

#### 3.4.1. Antibacterial and Antifungal Activity

**Figure 11:**
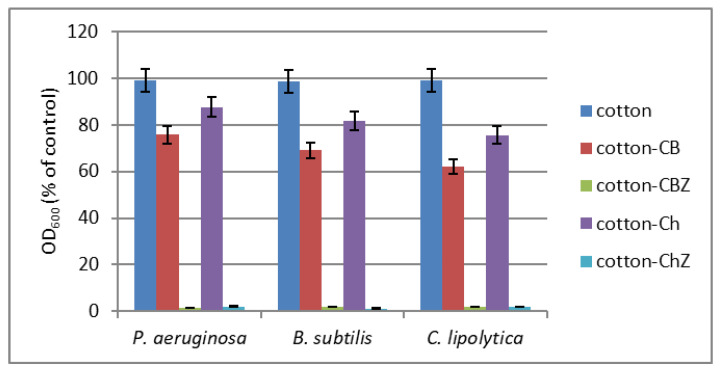
Growth of the test microbial strains in MPB liquid medium in the presence of untreated cotton fabric (control) and the CB and CBZ composites, compared to the Ch and ChZ samples obtained in a previous study.

The results of the antimicrobial studies of the CB and CBZ were compared with those of the virgin cotton fabric, used as a control, and with those of the Ch and ChZ obtained in a previous study [17]. In contrast to the newly obtained CB and CBZ, the commercial product chitosan was used for modification in the Ch and ChZ. The obtained results are presented in Figure 11. As the figure shows, all the composite materials exhibited antimicrobial activity in contrast to the pristine cotton fabric. The CBZ and ChZ samples containing zinc oxide particles completely inhibited the growth of all three model strains. In this case, the more pronounced hydrophobicity of the cotton materials containing zinc oxide prevented the bacteria from depositing on their surface and forming a biofilm. The CB was more active compared to the Ch, which was due to the modification of chitosan with benzaldehyde and to an increase in its hydrophobicity. The antimicrobial activity of the Ch and CB increased in the following order: fungal strain *C. Lipolytica* >Gram-positive bacteria *B. cereus* >Gram-negative bacteria *P. aeruginosa*.

#### 3.4.2. Virucidal Activity

The effectiveness of the CB and CBZ increased as the duration of interaction with the virus was extended. Within the first 30 min interval, a virucidal activity against the tested viruses was not observed. However, after 60 min of exposure, the samples began to exhibit virucidal activity. The CB showed a Δlog value of 0.1 against HRSV-2 and a Δlog value of 0.3 against HAdV-5 (refer to Table 4). The CBZ displayed a higher virucidal activity, with a Δlog value of 0.9 against HRSV-2 and a Δlog value of 0.9 against HAdV-5.

The virucidal tests involved carefully selected viruses with distinct biological characteristics: human adenovirus type 5 (HAdV5) and human respiratory syncytial virus (HRSV-S2). HRSV-S2 is an RNA virus that replicates in the cytoplasm, possessing a helical nucleocapsid enclosed in a bilipid layer that contains viral glycoproteins. It is vulnerable to pH and temperature changes and quickly loses its activity in acidic media, as well as in the presence of organic solvents such as ether, chloroform, etc. [30]. On the other hand, HAdV5 is a DNA virus, with replication in the cell nucleus, an icosahedral virus without an envelope, which makes it more resistant to chemical and physical methods designed to disrupt its structure in the environment [31].

## 4. Conclusions

Two new composite materials were obtained, based on cotton fabric, modified chitosan, with benzaldehyde deposited on them and additionally crosslinked with glutaraldehyde (material CB) and with in situ ZnO particles included in the chitosan film (material CBZ). Different analytical methods and microscopic techniques were used for their characterization. By optical microscopy, it was observed that a uniform chitosan layer was formed on the surface of cotton fabric. This coating was found to slow the thermal degradation process of the materials and prevent them from complete weight loss, as observed in the case of the untreated cotton fabric. The chemical modification of chitosan with benzaldehyde increased its hydrophobicity compared to the chitosan crosslinked only with glutaraldehyde, resulting in improved antimicrobial activity. Incorporation of ZnO particles into the layer resulted in the complete growth inhibition of all three model strains (the fungal strain C. lipolytica, the Gram-positive bacteria *B. cereus*, and the Gram-negative bacteria *P. aeruginosa*). The CB showed higher virucidal activity against the human adenovirus sero-type 5 (HAdV-5) than against the human respiratory syncytial virus (HRSV-S2) after 60 min of contact. It is assumed that in this case, the interaction with viruses is not only related to the destruction of the lipid envelope but to the binding and changing of the protein structure in the viral capsid. The presence of the ZnO particles in the chitosan film enhanced its virucidal activity against both enveloped and nonenveloped viruses. The results show that the approach used in the new composite materials preparation has the potential to achieve good microbiological and disinfection activity in textile materials.

## Figures and Tables

**Table 1:** Surface energy of the test solutions.

	Surface Energy (mN/m)		
Solutions	σ_1_	σ^d^_1_	σ^p^_1_
H_2_O	72.8	21.8	51.0
CH_2_I_2_	50.8	50.8	0

**Table 2:** Characteristic bands in the IR spectra for cellulose and chitosan.

Cellulose (cm^−1^)	Chitosan (cm^−1^)	Functional Groups
1256	w 1200	C-O-H
1150	1151	ν (C-O-C),
1125, 1110	1110	ν (CO), ν (CC), ring
1078	1080	ν (CO), ν (CC), ring
1034	1035	C-C-O, C-O-C, ring
895	895	β-glucoside bond

**Table 3:** Mean contact angle and free surface energy for water and diiodomethane on the CB and CBZ.

Sample	Contact Angle		Free Surface Energy (mN/m)		
	θ_H2O_ [°]	θ_CH2I2_ [°]	σ_s0_	σ^d^_s0_	σ ^p^_s0_
CB	77.13 ± 0.91	53.30 ± 2.10	38.72	32.41	6.31
CBZ	101.76 ± 1.14	51.45 ± 1.73	33.59	33.46	0.13

**Table 4:** The virucidal effect of CB and CBZ against the human respiratory syncytial virus (HRSV-S2) and human adenovirus C serotype 5 (HAdV-5) after 30 min/60 min.

Virus	Δlog 30 min		Δlog 60 min	
	CB	CBZ	CB	CBZ
HRSV-2	0	0	0.1	0.9
HAdV-5	0	0	0.3	0.9

## Data Availability

Data Availability Statements are available in section “MDPI Research Data Policies” at https://www.mdpi.com/ethics, accessed on 20 June 2023.
